# The differential effect of modern intravenous iron on fibroblast growth factor 23 and phosphate in non-dialysis dependent CKD – the exploratory randomized controlled double-blind ExplorIRON-CKD study

**DOI:** 10.1186/s12882-023-03440-7

**Published:** 2024-02-12

**Authors:** Xenophon Kassianides, Sunil Bhandari

**Affiliations:** Academic Renal Research Department, Hull University Teaching Hospitals NHS Trust and the Hull York Medical School, Kingston upon Hull, UK

**Keywords:** Bone metabolism, Chronic kidney disease, Ferric carboxymaltose, Ferric derisomaltose, Fibroblast growth factor 23, Intravenous iron, Iron deficiency, Phosphate, Vitamin D

## Abstract

**Background:**

Intravenous iron is commonly used in patients with non-dialysis-dependent chronic kidney disease (CKD). Modern intravenous iron compounds (e.g. ferric derisomaltose (FDI), ferric carboxymaltose (FCM)) are increasingly utilized with similar efficacy. A differential effect in terms of hypophosphatemia has been noted following administration of FCM, which may be related to fibroblast growth factor 23 (FGF23). This study was designed to examine the comparative effects of FDI and FCM on FGF23, phosphate and other markers of bone turnover.

**Methods:**

The single-center double-blind randomized controlled trial “Iron and Phosphaturia – ExplorIRON-CKD” primarily assessed the effects of FCM and FDI on intact FGF23 and phosphate, whilst also studying the impact on vitamin D, parathyroid hormone and phosphaturia. Bone markers including alkaline phosphatase, bone-specific alkaline phosphatase, procollagen type 1 N-terminal propeptide and carboxy-terminal collagen cross-linked telopeptide were monitored. Non-dialysis-dependent CKD patients (stage 3a-5) with iron deficiency with/without anemia (serum ferritin < 200 µg/L or transferrin saturation = 20% and serum ferritin 200-299 µg/L) were randomized to receive FDI or FCM in a 1:1 ratio. At baseline 1000 mg of intravenous iron was administered followed by 500-1000 mg at 1 month to achieve replenishment. Measurements were performed at baseline, 1–2 days following iron administration, 2 weeks, 1 month (second iron administration), 1–2 days following second administration, 2 months and 3 months following initial infusion.

**Results:**

Twenty-six patients participated in the trial; 14 randomized to FDI and 12 to FCM. Intact FGF23 increased following administration of iron, and the increase was significantly higher with FCM compared to FDI (Baseline to 1–2 days following 1st administration: FDI: 3.0 (IQR: - 15.1 - 13.8) % vs. FCM: 146.1 (IQR: 108.1–203.1) %; *p* < 0.001 and Baseline to 1–2 days following 2nd administration: FDI: 3.2 (IQR: - 3.5 – 25.4) % vs. FCM: 235.1 (138.5–434.6) %; *p* = 0.001). Phosphate levels decreased in the FCM group, causing a significant difference versus FDI 2 weeks following administration of the first dose. A significantly greater decrease in 1,25 (OH)_2_ Vitamin D was noted with FCM. Several markers of bone turnover significantly changed following administration of FCM but not FDI.

**Conclusions:**

The study suggests a differential effect on FGF23 following administration of FCM compared to FDI in non-dialysis-dependent CKD patients, similar to other patient groups. This may lead to changes consistent with hypovitaminosis D and alterations in bone turnover with potential clinical consequences. Further definitive studies are required to understand these differences of intravenous iron compounds.

**Trial registration:**

European Union Drug Regulating Authorities Clinical Trials Database (EudraCT) number: 2019–004370-26 (https://www.clinicaltrialsregister.eu/ctr-search/trial/2019-004370-26/GB) (First date of trial registration: 03/12/2019).

**Supplementary Information:**

The online version contains supplementary material available at 10.1186/s12882-023-03440-7.

## Background

Non-dialysis dependent chronic kidney disease (ND-CKD) has a number of complications, and their management is paramount in limiting the impact and progression of the disease. Iron deficiency anemia is a common complication with a prevalence ranging between 28 and 52%, increasing as kidney function deteriorates [1]. Current guidelines advocate alleviation of anemia using iron (oral or intravenous) [2, 3]. Nonetheless, given the chronic inflammatory status of ND-CKD limiting the utility and efficacy of oral iron, intravenous iron represents a suitable and effective alternative. Modern intravenous iron compounds (e.g. ferumoxytol, ferric carboxymaltose (FCM) and ferric derisomaltose (FDI)) have become increasingly used due to their ability to deliver higher doses of iron hence reducing visits to hospital and need for retreatment [4]. Studies within ND-CKD have highlighted the safety and efficacy of such compounds, with similar safety signals in terms of the traditional concerns pertaining to intravenous iron [5]. However, hypophosphatemia has been highlighted as much more common following FCM administration [6, 7]. Evidence from large-scale randomized controlled studies comparing FCM with other modern intravenous iron compounds in patients with iron deficiency anemia secondary to a multitude of causes, and more specifically due to inflammatory bowel disease have highlighted this association [8–11].

Hypophosphatemia was initially hypothesized to arise secondary to an increase in erythropoiesis due to iron repletion leading to increased phosphate uptake [6]. Further research into the mechanistic aspects behind this phenomenon identified fibroblast growth factor 23 (FGF23) as a key contributory factor [12]. Fibroblast growth factor 23 is a phosphatonin, with direct actions on phosphate metabolism in the body, leading to enhanced excretion of phosphate in the urine through inhibition of the sodium/phosphate (NaPi) type 2 cotransporters at the proximal convoluted tubule [13]. As FGF23 is intertwined with the absorption and excretion of this key mineral its effects extend to vitamin D, parathyroid hormone (PTH) and calcium. Ferric carboxymaltose and other intravenous iron compounds with similar carbohydrate moieties appear to halt the cleavage of the bioactive intact FGF23 (iFGF23) [14]. As such iFGF23 concentrations increase leading to the putative 6H syndrome described by Zoller and colleagues (high iFGF23, leading to hyperphosphaturia, hypophosphatemia, hypovitaminosis D, mild hypocalcemia and hyperparathyroidism) associated with iatrogenic intravenous iron induced hypophosphatemia [15]. Hypophosphatemia can be transient, however reports exist implicating hypophosphatemia acutely to rhabdomyolysis and heart failure, alongside osteomalacia and fractures in chronic cases, alongside symptoms of fatigue and muscle weakness [16, 17].

The differential effect of modern intravenous iron preparations has not been explored in ND-CKD. Evidence arising from randomized controlled trials comparing FCM and FDI have not included ND-CKD patients; indeed evidence of the 6H syndrome relevant to ND-CKD can only be extracted through observational studies. This is important due to the increasing use of such compounds in this population, and also the physiological differences that exist in FGF23 and phosphate metabolism in patients with ND-CKD when compared to patients with normal kidney function. Fibroblast growth factor 23 concentration increases as kidney function declines [18]. In addition, patients with ND-CKD frequently suffer from CKD-mineral bone disease characterized by abnormalities in the metabolism of calcium, phosphate, vitamin D and PTH leading to fractures and abnormalities in bone turnover [19]. Given the absence of research in this patient group, the “Iron and Phosphaturia – ExplorIRON-CKD” exploratory study was conducted to primarily explore the differential effect of two modern intravenous iron compounds (FDI vs. FCM) in patients with ND-CKD in terms of iFGF23. The differential effect on markers of the 6H syndrome was also secondarily examined, alongside biomarkers of bone turnover. Other secondary outcomes included hematinic response and impact on kidney function and inflammation.

## Methods

The “Iron and Phosphaturia – ExplorIRON-CKD” (EudraCT number: 2019–004370-26 / https://www.clinicaltrialsregister.eu/ctr-search/trial/2019-004370-26/GB/ first date of trial registration: 03/12/2019) was performed in accordance with Good Clinical Practice guidelines and the Declaration of Helsinki. The trial received the favorable opinion of the Health Research Authority and the Research Ethics Committee Leeds West (20/YH/0005), and clinical trial authorization by the Medicines and Healthcare Products Regulatory Agency of the United Kingdom. Study participants had all details explained to them in writing and in person before giving informed consent.

The methods have been previously published, but in brief this was an investigator led, exploratory single center double-blind randomized controlled trial designed to investigate the differential effect of FDI and FCM on intact FGF23 (iFGF23) and phosphate in patients with ND-CKD [20]. Patients with established ND-CKD (stages 3a-5) and serum ferritin < 200 µg/L and/or transferrin saturation = 20% and serum ferritin 200–299 µg/L were randomized in a 1:1 ratio to receive 1000 mg of FDI or FCM with a follow-up administration within 1 month of 500/1000 mg (visit 5) depending on weight and hematinic profile at baseline using a web-based application (www.sealedenvelope.com). The inclusion/exclusion criteria are further defined in Supplementary Table 1. The primary and secondary outcomes relevant to the study are presented in Supplementary Table 2.

Participants were enrolled in the study by the principal investigator who was blind to allocation. A single research nurse (who was not blinded) was responsible for assignment of patients and administration of medication. Participant and principal investigator/study doctor were blind to allocation throughout the study.

As this was an exploratory study looking for proof of concept and to be used for further hypothesis generation, no statistical power calculation took place. The study took place in a large tertiary teaching hospital in the United Kingdom.

### Measurements

Intact FGF23 (iFGF23), serum phosphate, serum calcium, PTH and vitamin D (in its active metabolic and inactive metabolite forms) were monitored. Fractional excretion of phosphate was calculated. Measurements were performed at baseline, 1–2 days following IV iron administration, 2 weeks, 1-month following administration (and second IV iron administration where suitable), 1–2 days following second administration and 2 months following initial infusion.

Markers of bone turnover (alkaline phosphatase (ALP), bone-specific ALP (BALP), procollagen type 1 N-terminal propeptide (P1NP) and carboxy-terminal collagen crosslinks (CTx)) were monitored within those pre-specified intervals. Hematinic response (hemoglobin, serum ferritin, transferrin saturation), kidney function/injury (serum creatinine, eGFR, urine protein creatinine ratio (PCR)) and inflammation (c-reactive protein) were also examined. Safety was assessed as per the obligations towards the regulatory authorities. Hypophosphatemia was defined as a value less than 0.65 mmol/L, and where present acted as a contra-indication for the second administration. Reference ranges for measured variables are indicated in Supplementary Table 3.

## 6H syndrome markers

Intact FGF23 was assessed using a chemiluminescence assay (Liaison XL, DiaSorin S.p.A., Saluggia, Italy). Serum phosphate and adjusted calcium were analyzed through the AU5800 automated analyser (Beckman Coulter, Nyon, Switzerland). 24-hour urinary phosphate excretion was also measured via the same technique, while fractional excretion of phosphate was calculated using the Walton and Bijvoet equation [21]. The Access Intact PTH assay (Beckman Coulter, California, USA), utilizing two-site immunoenzymatic technology was used for the measurement of PTH. Vitamin D, in terms of 1,25(OH)_2_ Vitamin D was measured using a chemiluminescence assay (Liaison XL, DiaSorin S.p.A., Saluggia, Italy), while metabolites were assessed through liquid chromatography and tandem mass spectrometry previously described by Tang and colleagues [22].

## Bone turnover markers

Alkaline phosphatase was assessed through the AU5800 automated analyzer. Bone specific ALP was measured through enzyme-linked immunosorbent assays (ELISA) provided by Quidel (Quidel, San Diego, California USA). Procollagen type 1 N-terminal pro-peptide and CTx were also assessed using ELISA technology (Roche Diagnostics, Risch-Rotkreuz, Switzerland).

## Hematinic response

Hemoglobin, serum ferritin and transferrin saturation were analyzed using automated processes. The SYSMEX XN-9100 (Sysmex Corporation, Kobe, Japan) was used for the measurement of hemoglobin. Beckman Coulter technology was employed in the analysis of serum ferritin (two-site immunoenzymatic assay) and transferrin saturation (AU5800 automated analyzer).

## Kidney function/injury

An automated enzymatic assay performed by the AU5800 was used in the measurement of creatinine (serum and urinary). Estimated glomerular filtration rate was calculated using the CKD-EPI calculation 2009. Urinary protein: creatinine ratio as a surrogate marker of kidney injury was calculated using the measurements of urinary protein and creatinine reported through automated procedure by AU5800.

## Inflammation

C-reactive protein was measured using the AU5800 analyzer.

### Statistical analysis

An intention-to-treat approach was adopted, with all randomized participants included in the statistical analysis. Continuous data is presented as mean (standard deviation) or median (interquartile range) depending on normality of data distribution. Categorical data is summarized as number and percentage (%). Normality of distribution was assessed using the Shapiro-Wilk test. As per prespecified outcomes between groups analysis (i.e. FDI vs. FCM) was conducted using independent T-test and Mann-Whitney U test depending on data distribution. Percentage change was also used to assess change between variables relevant to the 6H syndrome; groups were compared using the aforementioned statistical tests. The Fisher’s exact test was used to detect differences between the two groups in terms of categorical data. The Skillings-Mack test was performed to identify any within-group trends in terms of concentration. The combined effect of iron supplementation in terms of iFGF23 and phosphate was based on previous work by Huang and colleagues and Stohr and colleagues in patients with ND-CKD receiving FCM [23, 24]. In these studies maximum %change iFGF23 was noted on day 2 (248% (*p* < 0.0001) and 80% (*p* > 0.05)) and maximum negative %change in phosphate was seen on days 7 and 14 respectively (- 23% (*p* < 0.001) and - 20% (*p* > 0.50)). Given these values and the expected trajectory of iFGF23 and phosphate, the limits of either > 200% change in iFGF23 and/or > - 20% in phosphate concentration were set up in the present study. The combined effect of iron supplementation as composite of change was examined using % change of iFGF23 from baseline and % change in serum phosphate from baseline to day 2 and week 2 (i.e. visits 3 and 4). This was an exploratory outcome and no statistical analysis was performed. A statistical software package was used to perform the analysis (IBM SPSS Statistics Version 26, IBM Corp. 2019). Statistical significance was inferred from a *p*-value < 0.05.

## Results

### Baseline

**Fig. 1:**
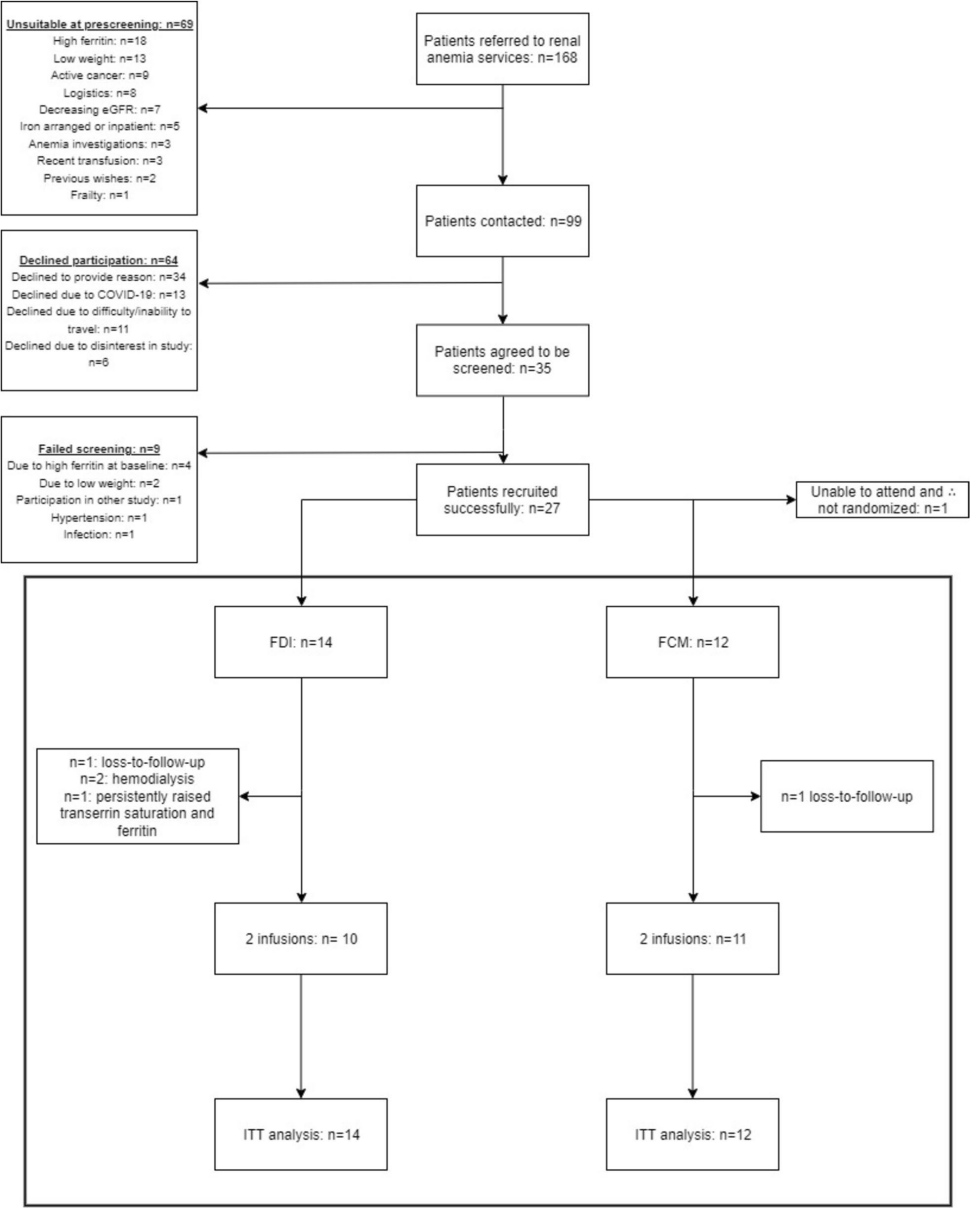
Consort diagram 168 patients were referred to the kidney anemia services and pre-screened; 99 of those fulfilled in principle the eligibility criteria and were contacted. 35 patients accepted to be screened, and 27 were eligible for the trial. One participant withdrew from the trial prior to randomization

The consort diagram (Fig. 1) displays the patient flow from pre-screening to end of trial. The study ran between March 2020 and July 2021.A total of 26 patients were randomized to receive either FDI (*n* = 14) or FCM (*n* = 12). The majority of the participants in the study were male (65%) and all of them of white British origin. At baseline the two groups were comparable for all variables assessed with the exception of age, 24-hour urinary phosphate excretion and heart failure incidence (Tables 1 and 2). There was no statistically significant difference between markers of 6H, including fractional excretion of phosphate. Most participants had iron deficiency with anemia (*n* = 24; 92.3%; FCM: 91.7% vs. FDI: 92.9%) and CKD stage 4 (eGFR 15–30 ml/min/1.73m^2^).

**Table 1:** Baseline continuous variable values in both groups and total

Variable	Iron group	Value	*p*-value
Age* / years	Total	67.9 (12.4)	0.043
	FDI	63.4 (12.2)	
	FCM	73.2 (10.8)	
Body mass index / kg/m^2^	Total	27.8 (25.0–33.4)	0.279
	FDI	28.8 (26.2–36.3)	
	FCM	26.8 (23.0–32.7)	
iFGF23 / pg/ml	Total	212.1 (145.1–311.6)	0.212
	FDI	257.3 (136.2–584.6)	
	FCM	186.5 (143.6–226.6)	
Phosphate / mmol/L	Total	1.28 (1.12–1.43)	0.193
	FDI	1.30 (1.16–1.59)	
	FCM	1.20 (1.07–1.38)	
Hemoglobin* / g/L	Total	100.3 (13.5)	0.664
	FDI	99.2 (12.2)	
	FCM	101.6 (15.3)	
Serum Ferritin / µg/L	Total	76.5 (38.7–157.5)	0.899
	FDI	76.5 (25.0–183.5)	
	FCM	72.7 (42.3–146.9)	
Transferrin saturation / %	Total	15.0 (11.7–18.5)	0.781
	FDI	15.0 (11.0–21.0)	
	FCM	14.5 (12.0–17.8)	
Creatinine* / µmol/L	Total	269.5 (88.2)	0.626
	FDI	277.6 (98.8)	
	FCM	260.2 (77.3)	
eGFR / ml/min/1.73m^2^	Total	18.0 (14.0–25.3)	1.000
	FDI	18.0 (14.0–25.3)	
	FCM	18.0 (14.0–25.3)	
CRP / mg/L	Total	7.4 (3.3–17.3)	0.462
	FDI	8.0 (3.2–20.8)	
	FCM	4.3 (3.4–13.4)	
urinary PCR / mg/mmol	Total	87.5 (30.0–341.3)	0.082
	FDI	155.0 (57.5–607.5)	
	FCM	30.0 (20.0–310.0)	
1,25 (OH)2 Vitamin D* / pmol/L	Total	45.6 (22.2)	0.290
	FDI	41.3 (20.8)	
	FCM	50.7 (23.5)	
25 (OH)2 Vitamin D / nmol/L	Total	57.4 (22.4–86.0)	0.252
	FDI	44.2 (18.3–83.1)	
	FCM	67.5 (29.9–97.0)	
24(R),25 (OH)2 Vitamin D / nmol/L	Total	2.2 (0.6–3.4)	0.631
	FDI	1.2 (0.5–4.5)	
	FCM	2.9 (0.9–3.2)	
Calcium* / mmol/L	Total	2.35 (0.08)	0.813
	FDI	2.35 (0.08)	
	FCM	2.34 (0.09)	
PTH / pmol/L	Total	17.4 (11.3–22.6)	0.145
	FDI	18.9 (12.9–28.8)	
	FCM	16.3 (7.9–20.9)	
24 hr. urinary phosphate / mmol	Total	17.5 (11.0–22.3)	0.023
	FDI	21.0 (15.0–23.5)	
	FCM	12.5 (10.3–17.8)	
Fractional excretion of phosphate/ %	Total	43.2 (32.9–55.6)	0.374
	FDI	49.7 (32.2–58.3)	
	FCM	36.4 (32.9–54.5)	
ALP / [iU]/L	Total	97.0 (79.0–144.3)	0.667
	FDI	96.0 (79.0–153.0)	
	FCM	107.0 (77.8–129.8)	
BALP / [U]/L	Total	19.5 (15.0–26.4)	0.462
	FDI	21.3 (16.6–26.8)	
	FCM	18.7 (14.1–27.5)	
CTx / µg/ml	Total	0.89 (0.52–1.07)	0.560
	FDI	0.84 (0.56–1.01)	
	FCM	0.98 (0.49–1.18)	
P1NP / µg/L	Total	103.0 (63.0–174.3)	0.820
	FDI	112.0 (70.3–178.8)	
	FCM	103.0 (63.0–166.3)	

Variables noted with *: data presented as mean (SD); otherwise data is presented as median (IQR)

*p*-value represents the statistical significant upon comparison of two groups

**Table 2:** Baseline categorical variable values

Variable	Total	FDI	FCM	*P*-value
Gender				
Male	17 (65.3)	8 (57.1)	9 (75.0)	
Female	9 (34.6)	6 (42.8)	3 (25.0)	0.429
Smoking status				
Smoker	7 (27.0)	5 (35.7)	2 (16.7)	
Ex-smoker	14 (53.8)	5 (35.7)	9 (75.0)	
Non-smoker	5 (19.2)	4 (28.6)	1 (8.3)	N/A
CKD stage				
3b	3 (11.5)	1 (7.1)	2 (16.7)	
4	16 (61.5)	9 (64.3)	7 (58.3)	
5	7 (26.9)	4 (28.6)	3 (25.0)	N/A
Ethnicity				
White	26 (100.0)	14 (100.0)	12 (100.)	
Black	0 (0.0)	0 (0.0)	0 (0.0)	
Other	0 (0.0)	0 (0.0)	0 (0.0)	N/A
Hypertension	21 (80.8)	13 (92.9)	8 (66.7)	0.148
Type I Diabetes Mellitus	2 (7.7)	2 (14.3)	0 (0.0)	0.483
Type II Diabetes Mellitus	11 (42.3)	6 (42.8)	5 (41.7)	1.000
Heart failure	7 (26.9)	1 (7.1)	6 (50.0)	0.0026
Ischemic Heart disease	10 (38.5)	4 (28.6)	6 (50.0)	0.422
Previous cancer	5 (19.2)	2 (14.3)	3 (25.0)	0.635
Medications				
Erythropoiesis stimulating agents	8 (30.8)	5 (35.7)	3 (25.0)	0.683
Renin-angiotensin-aldosterone system associated medications	16 (61.5)	9 (64.3)	7 (58.3)	1.000
Vitamin D supplementation	6 (23.1)	4 (28.6)	2 (16.7)	0.652
Diuretics	14 (53.8)	7 (50.0)	7 (58.3)	0.713
ß-blockers	18 (69.2)	9 (64.3)	9 (75.0)	0.683
Cause				
Autosomal Dominant Polycystic Kidney Disease	1 (3.8)	1 (7.1)	0 (0.0)	N/A
Multifactorial	6 (23.1)	2 (14.3)	4 (33.3)	N/A
Primary renovascular	2 (7.7)	1 (7.1)	1 (8.3)	N/A
Glomerulosclerosis	1 (3.8)	0 (0.0)	1 (8.3)	N/A
Unknown	1 (3.8)	1 (7.1)	0 (0.0)	N/A
Diabetic nephropathy	3 (11.5)	2 (14.3)	1 (8.3)	N/A
IgA nephropathy	2 (7.7)	1 (7.1)	1 (8.3)	N/A
Nephrectomy	1 (3.8)	0 (0.0)	1 (8.3)	N/A
Kidney aplasia and obstructive uropathy	1 (3.8)	1 (7.1)	0 (0.0)	N/A
Chronic pyelonephritis	1 (3.8)	1 (7.1)	0 (0.0)	N/A
Membranous nephropathy	3 (11.5)	3 (21.4)	0 (0.0)	N/A
Cardiorenal syndrome	2 (7.7)	0 (0.0)	2 (16.7)	N/A
Hypertension	1 (3.8)	1 (7.1)	0 (0.0)	N/A
Systemic Lupus Erythematosus	1 (3.8)	0 (0.0)	1 (8.3)	N/A

All participants received at least one dose of intravenous iron, and 21 of the 26 received a second dose as per protocol. The mean dose of iron administered was 1428.6 (SD: 331.5) mg and 1500 (SD: 213.2) mg in the FDI and FCM groups respectively (*p* = 0.53). The on-going COVID-19 pandemic limited the recruitment to a full pre-designed number of 30.

### Intact FGF23 and phosphate

**Fig. 2:**
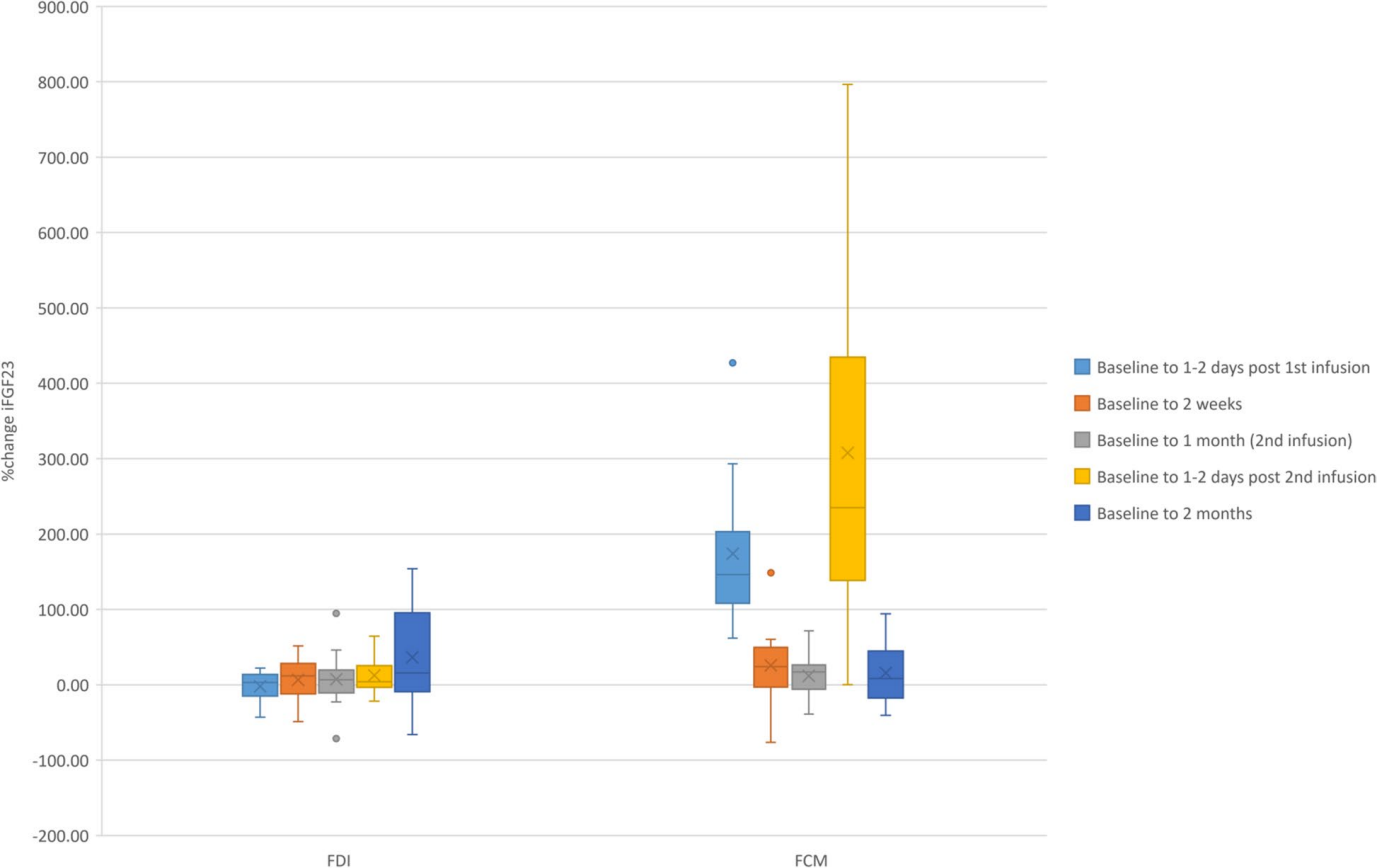
Box plots representation of %change in iFGF23 following intervention with FDI or FCM. Legend: %change iFGF23 following infusion with FDI or FCM. Statistically significant larger %change (increase) where FCM was infused, between baseline and 1–2 days following 1st infusion and baseline and 2nd infusion. This was reflected in the significantly higher iFGF23 concentration 1–2 days following 2nd infusion in those administered FCM when compared to those receiving FDI. Dots represent outliers

Intact FGF23 concentrations were not significantly different from baseline concentrations in the FDI group throughout the trial. A maximum increase 1–2 days post administration the first dose of FCM and later at 1–2 days following administration of the second IV iron dose was noted. This resulted in a statistically significantly greater % change in iFGF23 compared to FDI (FDI: 3.0 (IQR: - 15.1 - 13.8) % vs. FCM: 146.1 (IQR: 108.1–203.1) %; *p* < 0.001 and FDI: 3.2 (IQR: - 3.5 – 25.4) % vs. FCM: 235.1 (138.5–434.6) %; *p* = 0.001) respectively (Fig. 2) (Table 3).

**Table 3:** % change in terms of variables relevant to the 6H syndrome

Variable	Iron group (n)	Mean/Median (SD/IQR)	*p*-value
**iFGF23**			
Baseline to 1–2 days after 1st infusion	FDI (14)	3.0 (28.9)	
	FCM (11)	146.1 (94.9)	< 0.001
Baseline to 2 weeks after 1st infusion	FDI (13)	11.9 (40.6)	
	FCM (10)	24.3 (52.7)	0.284
Baseline to 1 month after 1st infusion (2nd infusion)	FDI (12)	6.5 (30.3)	
	FCM (11)	17.1 (32.3)	0.566
Baseline to 1–2 days after 2nd infusion	FDI (9)	3.2 (28.9)	
	FCM (10)	235.1 (296.1)	0.001
Baseline to 2 months after 1st infusion	FDI (12)	15.7 (104.7)	
	FCM (10)	8.1 (62.1)	0.497
**Phosphate**			
Baseline to 1–2 days after 1st infusion	FDI (14)	-6.5 (15.1)	
	FCM (11)	-3.3 (18.2)	0.893
Baseline to 2 weeks after 1st infusion	FDI (13)	-1.6 (20.6)	
	FCM (10)	-11.0 (17.8)	0.077
Baseline to 1 month after 1st infusion (2nd infusion)	FDI (12)	-7.5 (25.1)	
	FCM (11)	-6.1 (14.3)	1.000
Baseline to 1–2 days after 2nd infusion	FDI (9)	1.8 (30.3)	
	FCM (10)	-14.9 (14.7)	0.013
Baseline to 2 months after 1st infusion	FDI (13)	9.2 (25.1)	
	FCM (10)	-13.2 (18.5)	0.131
**Calcium** ^**a**^			
Baseline to 1–2 days after 1st infusion	FDI (14)	1.5 (2.7)	
	FCM (11)	0.4 (2.5)	0.297
Baseline to 2 weeks after 1st infusion	FDI (13)	0.4 (3.4)	
	FCM (10)	-1.8 (3.1)	0.131
Baseline to 1 month after 1st infusion (2nd infusion)	FDI (12)	0.5 (2.9)	
	FCM (11)	0.3 (3.8)	0.916
Baseline to 1–2 days after 2nd infusion	FDI (9)	1.5 (2.2)	
	FCM (10)	-1.0 (2.4)	0.035
Baseline to 2 months after 1st infusion	FDI (12)	0.5 (4.7)	
	FCM (10)	1.5 (3.2)	0.566
**Fractional excretion of phosphate**			
Baseline to 1–2 days after 1st infusion	FDI (11)	-7.1 (26.2)	
	FCM (10)	6.4 (27.9)	0.314
Baseline to 2 weeks after 1st infusion	FDI (12)	-3.7 (18.1)	
	FCM (10)	-5.0 (28.9)	0.872
Baseline to 1 month after 1st infusion (2nd infusion)	FDI (9)	-2.7 (13.0)	
	FCM (10)	3.6 (33.9)	0.661
Baseline to 1–2 days after 2nd infusion	FDI (9)	-8.2 (31.6)	
	FCM (10)	13.0 (48.4)	0.182
Baseline to 2 months after 1st infusion	FDI (10)	5.0 (24.8)	
	FCM (10)	0.3 (28.0)	1.000
**1,25 (OH)**_**2**_ **Vitamin D** ^**a**^			
Baseline to 1–2 days after 1st infusion	FDI (14)	-2.8 (14.2)	
	FCM (11)	-15.6 (12.8)	0.027
Baseline to 2 weeks after 1st infusion	FDI (13)	-14.7 (14.6)	
	FCM (10)	-18.1 (14.9)	0.580
Baseline to 1 month after 1st infusion (2nd infusion)	FDI (12)	-9.8 (16.5)	
	FCM (11)	-5.4 (27.2)	0.642
Baseline to 1–2 days after 2nd infusion	FDI (9)	-4.7 (13.0)	
	FCM (10)	-24.9 (22.5)	0.031
Baseline to 2 months after 1st infusion	FDI (12)	-5.4 (30.5)	
	FCM (10)	-5.3 (29.1)	0.933
**25 (OH)**_**2**_ **Vitamin D**			
Baseline to 1–2 days after 1st infusion	FDI (14)	1.6 (14.0)	
	FCM (11)	-3.5 (17.5)	0.267
Baseline to 2 weeks after 1st infusion	FDI (13)	5.8 (17.7)	
	FCM (10)	-0.3 (10.6)	0.483
Baseline to 1 month after 1st infusion (2nd infusion)	FDI (12)	-11.0 (28.8)	
	FCM (11)	-7.1 (26.0)	0.880
Baseline to 1–2 days after 2nd infusion	FDI (9)	0.8 (20.0)	
	FCM (10)	-8.5 (21.7)	0.549
Baseline to 2 months after 1st infusion	FDI (12)	-1.0 (48.8)	
	FCM (10)	-3.0 (25.7)	0.923
**24(R), 25 (OH)**_**2**_ **Vitamin D**			
Baseline to 1–2 days after 1st infusion	FDI (14)	0.0 (7.7)	
	FCM (11)	-8.3 (14.3)	0.085
Baseline to 2 weeks after 1st infusion	FDI (13)	0.0 (7.3)	
	FCM (10)	12.0 (21.1)	0.067
Baseline to 1 month after 1st infusion (2nd infusion)	FDI (12)	-4.2 (29.3)	
	FCM (11)	13.5 (37.5)	0.695
Baseline to 1–2 days after 2nd infusion	FDI (9)	-3.0 (21.6)	
	FCM (10)	6.3 (27.8)	0.842
Baseline to 2 months after 1st infusion	FDI (12)	0.0 (48.4)	
	FCM (10)	-4.1 (26.8)	0.923
**PTH** ^**a**^			
Baseline to 1–2 days after 1st infusion	FDI (14)	-7.8 (22.0)	
	FCM (11)	-6.9 (25.4)	0.927
Baseline to 2 weeks after 1st infusion	FDI (13)	2.9 (30.8)	
	FCM (10)	19.6 (32.9)	0.226
Baseline to 1 month after 1st infusion (2nd infusion)	FDI (12)	-12.1 (32.0)	
	FCM (11)	13.6 (27.7)	0.054
Baseline to 1–2 days after 2nd infusion	FDI (9)	-3.7 (29.5)	
	FCM (10)	-5.0 (24.3)	0.913
Baseline to 2 months after 1st infusion	FDI (12)	1.8 (36.7)	
	FCM (10)	-1.6 (28.1)	0.810

^a^ variables characterised by asterisk are described as mean (SD); the remaining variables are described as median (IQR) based on distribution

**Fig. 3:**
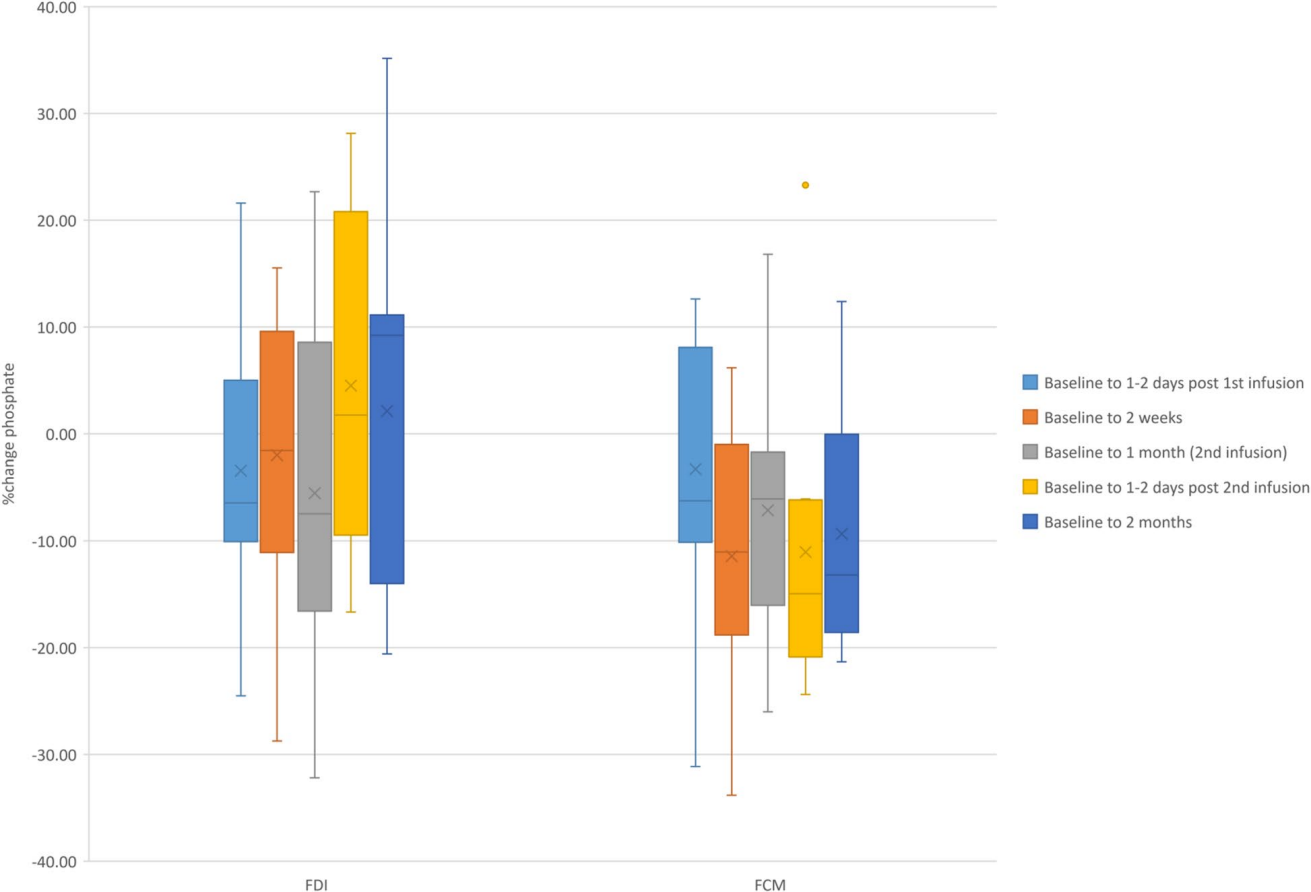
Box plots representation of %change in phosphate concentration following intervention with FDI or FCM. Legend: %change phosphate following infusion with FDI or FCM. A statistically significant differential effect was noted between baseline and 1–2 days following 2nd infusion, whereby FCM was associated with a greater decrease at that visit. Dots represent outliers

Phosphate concentrations at baseline were not significantly different between FDI and FCM, however they reduced following FCM leading to a significant % change between baseline and 1–2 days post 2nd iron administration (FDI: 1.8 (IQR: - 9.5 – 20.8) % vs. FCM: -14.9 (IQR: - 20.9 - -6.2)%; *p* = 0.013) (Fig. 3) (Table 3). A significant difference was observed in phosphate concentration at 2 weeks (FDI: 1.26 (IQR: 1.05–1.66) mmol/L vs. FCM: 1.09 (IQR: 0.94–1.23) mmol/L; *p* = 0.049) (Table 4). No participant in the FDI group developed an increase in iFGF23 > 200% throughout the study, whilst this was evident in 8 (66.7%) participants receiving FCM group, with three developing a further decrease in phosphate defined as a decrease > 20%.

**Table 4:** Concentrations of variables relevant to the 6H syndrome

Visit	Iron group (n)	Mean/Median (SD/IQR)	*p*-value	*p*- value (within group analysis)
**iFGF23**				
Baseline	FDI (14)	257.3 (136.2–584.6) pg/ml	0.212	
	FCM (12)	186.5 (143.6–226.6) pg/ml		
1–2 days post 1st infusion	FDI (14)	251.0 (156.6–583.0) pg/ml	0.066	
	FCM (11)	467.1 (334.6–655.7) pg/ml		
2 weeks	FDI (13)	233.6 (161.0–417.6) pg/ml	0.410	
	FCM (10)	199.7 (144.4–329.1) pg/ml		
1 month (2nd infusion)	FDI (12)	226.5 (137.2–428.9) pg/ml	0.487	
	FCM (11)	212.1 (116.0–253.7) pg/ml		
1–2 days post 2nd infusion	FDI (9)	262.4 (129.1–468.1) pg/ml	0.035	
	FCM (10)	662.7 (375.9–1633.8) pg/ml		
2 months	FDI (12)	301.2 (144.4–533.9) pg/ml	0.497	Within FDI: 0.058
	FCM (10)	227.8 (165.3–285.3) pg/ml		Within FCM 0.001
**Phosphate**				
Baseline	FDI (14)	1.30 (1.16–1.59) mmol/L	0.193	
	FCM (12)	1.20 (1.07–1.38) mmol/L		
1–2 days post 1st infusion	FDI (14)	1.37 (1.12–1.58) mmol/L	0.647	
	FCM (11)	1.23 (1.04–1.35) mmol/L		
2 weeks	FDI (13)	1.26 (1.05–1.66) mmol/L	0.049	
	FCM (10)	1.09 (0.94–1.23) mmol/L		
1 month (2nd infusion)	FDI (12)	1.18 (1.07–1.57) mmol/L	0.449	
	FCM (11)	1.14 (0.95–1.31) mmol/L		
1–2 days post 2nd infusion	FDI (9)	1.23 (1.15–1.66) mmol/L	0.065	
	FCM (10)	1.11 (0.91–1.36) mmol/L		
2 months	FDI (13)	1.33 (1.13–1.67) mmol/L	0.057	Within FDI: 0.278
	FCM (10)	1.13 (1.00–1.22) mmol/L		Within FCM: 0.129
**Calcium** ^**a**^				
Baseline	FDI (14)	2.35 (0.08) mmol/L	0.813	
	FCM (12)	2.34 (0.09) mmol/L		
1–2 days post 1st infusion	FDI (14)	2.39 (0.11) mmol/L	0.286	
	FCM (11)	2.34 (0.10) mmol/L		
2 weeks	FDI (13)	2.36 (0.11) mmol/L	0.123	
	FCM (10)	2.29 (0.06) mmol/L		
1 month (2nd infusion)	FDI (12)	2.35 (0.09) mmol/L	0.698	
	FCM (11)	2.32 (0.08) mmol/L		
1–2 days post 2nd infusion	FDI (9)	2.38 (0.10) mmol/L	0.063	
	FCM (10)	2.31 (0.06) mmol/L		
2 months	FDI (13)	2.35 (0.11) mmol/L	0.807	Within FDI: 0.473
	FCM (10)	2.36 (0.07) mmol/L		Within FCM: 0.544
**Fractional excretion of phosphate**				
Baseline	FDI (14)	49.7 (32.2–58.3) %	0.374	
	FCM (12)	36.4 (32.9–54.5) %		
1–2 days post 1st infusion	FDI (11)	41.8 (31.4–51.5) %	0.918	
	FCM (10)	41.3 (34.9–50.1) %		
2 weeks	FDI (12)	41.9 (30.2–47.8) %	0.722	
	FCM (10)	40.8 (28.8–53.6) %		
1 month (2nd infusion)	FDI (9)	40.3 (30.1–53.1) %	0.968	
	FCM (10)	42.6 (28.2–53.6) %		
1–2 days post 2nd infusion	FDI (8)	40.1 (27.2–50.2) %	0.897	
	FCM (10)	40.3 (28.2–50.7) %		
2 months	FDI (10)	48.3 (28.5–54.8) %	0.631	Within FDI: 0.927
	FCM (10)	42.2 (31.0–47.4) %		Within FCM: 0.412
**1,25 (OH)**_**2**_ **Vitamin D** ^**a**^				
Baseline	FDI (14)	41.3 (20.8) pmol/L	0.290	
	FCM (12)	50.7 (23.5) pmol/L		
1–2 days post 1st infusion	FDI (14)	41.0 (25.6) pmol/L	0.702	
	FCM (11)	44.8 (21.9) pmol/L		
2 weeks	FDI (13)	37.5 (18.6) pmol/L	0.841	
	FCM (10)	39.0 (15.1) pmol/L		
1 month (2nd infusion)	FDI (12)	40.0 (20.5) pmol/L	0.398	
	FCM (11)	47.5 (20.9) pmol/L		
1–2 days post 2nd infusion	FDI (9)	45.8 (42.2) pmol/L	0.283	
	FCM (10)	36.2 (12.6) pmol/L		
2 months	FDI (12)	41.9 (22.2) pmol/L	0.491	Within FDI: 0.264
	FCM (10)	49.0 (25.3) pmol/L		Within FCM: 0.026
**25(OH)**_**2**_ **Vitamin D**				
Baseline	FDI (14)	44.2 (18.3–83.1) nmol/L	0.252	
	FCM (12)	67.5 (29.9–97.0) nmol/L		
1–2 days post 1st infusion	FDI (14)	44.9 (18.7–83.7) nmol/L	0.467	
	FCM (11)	54.5 (22.1–87.1) nmol/L		
2 weeks	FDI (13)	45.2 (17.3–90.8) nmol/L	0.832	
	FCM (10)	69.0 (22.2–89.8) nmol/L		
1 month (2nd infusion)	FDI (12)	35.7 (15.9–83.7) nmol/L	0.235	
	FCM (11)	68.1 (26.3–102.1) nmol/L		
1–2 days post 2nd infusion	FDI (9)	57.8 (20.5–88.3) nmol/L	0.604	
	FCM (10)	63.3 (25.5–97.0) nmol/L		
2 months	FDI (12)	47.4 (19.2–93.2) nmol/L	0.346	Within FDI: 0.945
	FCM (10)	70.2 (28.9–112.3) nmol/L		Within FCM: 0.977
**24(R),25 (OH)2 Vitamin D**				
Baseline	FDI (14)	1.2 (0.5–4.5) nmol/L	0.631	
	FCM (12)	2.9 (0.9–3.2) nmol/L		
1–2 days post 1st infusion	FDI (14)	1.2 (0.6–4.2) nmol/L	0.727	
	FCM (11)	2.5 (0.8–3.3)) nmol/L		
2 weeks	FDI (13)	1.2 (0.6–4.3) nmol/L	0.693	
	FCM (10)	3.1 (0.7–3.9) nmol/L		
1 month (2nd infusion)	FDI (12)	1.0 (0.5–3.6) nmol/L	0.347	
	FCM (11)	2.6 (0.7–3.8) nmol/L		
1–2 days post 2nd infusion	FDI (9)	1.7 (0.7–4.1) nmol/L	1.000	
	FCM (10)	2.9 (0.9–3.6) nmol/L		
2 months	FDI (12)	1.3 (0.5–4.8) nmol/L	0.539	Within FDI: 0.902
	FCM (10)	2.8 (0.9–3.5) nmol/L		Within FCM: 0.406
**PTH**				
Baseline	FDI (14)	18.9 (12.9–28.8) pmol/L	0.145	
	FCM (12)	16.3 (7.9–20.9) pmol/L		
1–2 days post 1st infusion	FDI (14)	16.2 (10.8–27.9) pmol/L	0.344	
	FCM (11)	13.2 (7.2–18.8) pmol/L		
2 weeks	FDI (13)	19.6 (8.3–34.5) pmol/L	0.564	
	FCM (10)	17.2 (10.5–20.1) pmol/L		
1 month (2nd infusion)	FDI (12)	18.2 (7.2–28.4) pmol/L	0.651	
	FCM (11)	17.5 (9.6–22.6) pmol/L		
1–2 days post 2nd infusion	FDI (9)	14.4 (7.4–25.8) pmol/L	0.604	
	FCM (10)	13.4 (7.6–17.5) pmol/L		
2 months	FDI (12)	20.2 (8.9–22.1) pmol/L	0.283	Within FDI: 0.299
	FCM (10)	12.8 (8.1–20.1) pmol/L		Within FCM: 0.081

^a^ variables characterized by asterisk are described as mean (SD), the remaining variables are described as median (IQR) based on distribution

No moderate or severe hypophosphatemia were noted as per protocol (serum phosphate concentration < 0.65 mmol/L). There was no postponement of second infusion due to hypophosphatemia.

### Other markers of 6H syndrome

**Fig. 4:**
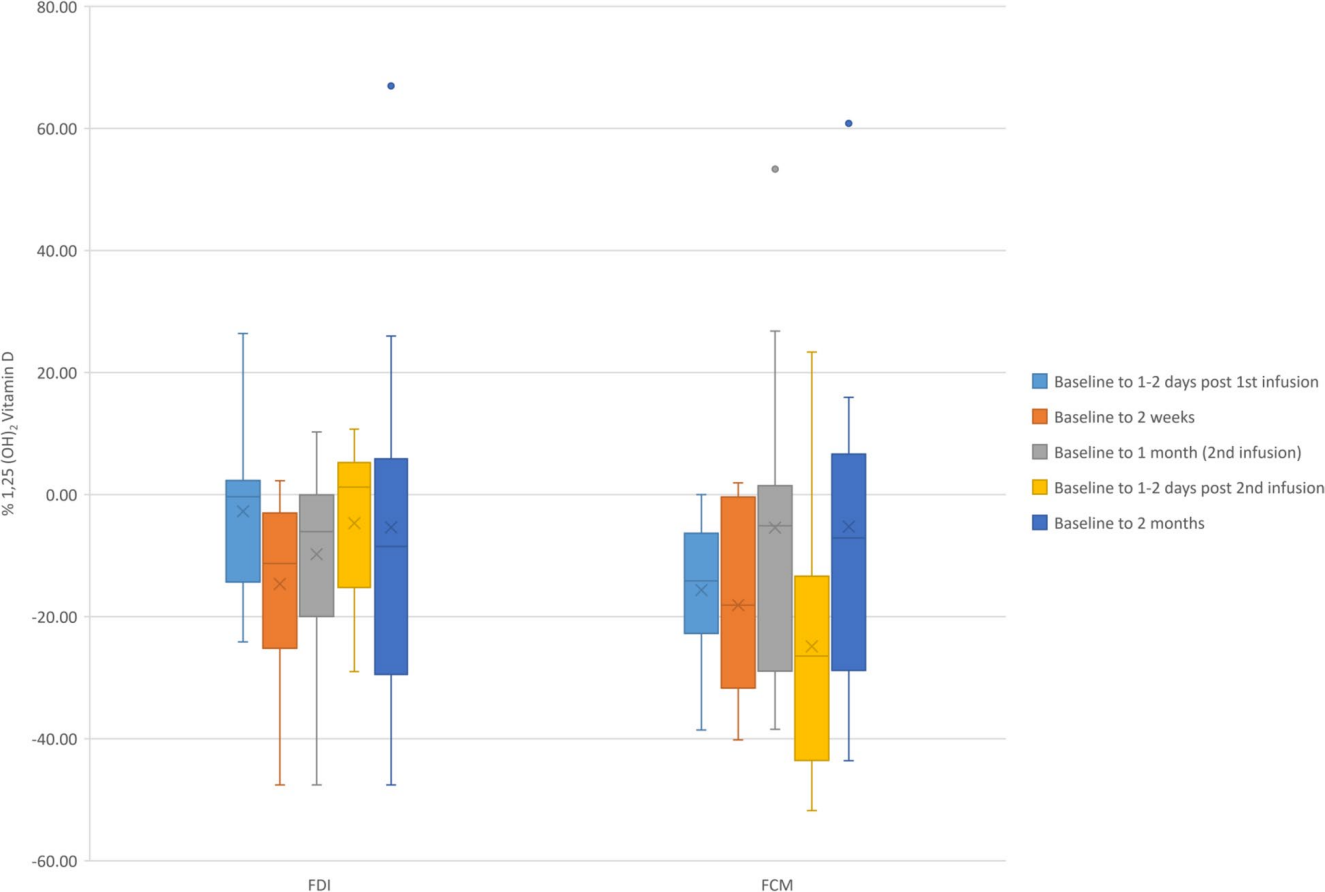
Box plots representation of %change in 1,25 (OH)_2_ Vitamin D following intervention. Legend: % change in 1,25 (OH)_2_ Vitamin D following infusion with FDI or FCM. A statistically significant differential effect is noted between baseline and 1–2 days post 1st and 2nd infusion following initial administration. FCM was associated with a greater decrease following both the 1st and 2nd infusion. Dots represent outliers

There was a significantly greater % reduction in 1,25 (OH)_2_ Vitamin D for the FCM group compared with the FDI group from baseline 1–2 days following first infusion (*p* = 0.027) and 1–2 days following second infusion (*p* = 0.031) (Fig. 4). Vitamin D metabolites (including 25 (OH)_2_ Vitamin D and 24(R), 25 (OH)_2_ Vitamin D) were similar in both groups. The % change calcium after 1–2 days of administration of the second dose of iron was greater for FDI (FDI: 1.5 (SD: 2.2) % vs. FCM: -1.0 (SD: 2.4) %; *p* = 0.035); calcium concentration remained within the normal range (Table 3). PTH and markers of phosphaturia were similar in both groups throughout the trial (Table 4).

### Bone turnover markers

Ferric carboxymaltose was associated with a significant difference between concentrations from baseline of ALP (*p* = 0.016), BALP (*p* < 0.001) and CTx (*p* = 0.006). No differential effect was noted throughout the study. Table 5 summarizes the results of the study on bone turnover markers.

**Table 5:** Concentrations of markers of bone turnover

Visit	Iron group (n)	Median (IQR)	*p*-value	*p*- value (within group analysis)
**ALP**				
Baseline	FDI (14)	96.0 (79.0–153.0) [iU]/L	0.667	
	FCM (12)	107.0 (77.8–129.8) [iU]/L		
1–2 days post 1st infusion	FDI (14)	104.0 (82.5–159.5) [iU]/L	0.893	
	FCM (11)	110.0 (82.0–150.0) [iU]/L		
2 weeks	FDI (13)	110.0 (74.0–136.5) [iU]/L	0.832	
	FCM (10)	112.5 (90.5–139.0) [iU]/L		
1 month (2nd infusion)	FDI (12)	99.5 (78.2–131.5) [iU]/L	0.525	
	FCM (11)	112.0 (90.0–140.0) [iU]/L		
1–2 days post 2nd infusion	FDI (9)	110.0 (78.0–136.0) [iU]/L	0.604	
	FCM (10)	123.5 (87.5–152.8) [iU]/L		
2 months	FDI (13)	124.0 (90.0–145.0) [iU]/L	0.879	Within FDI: 0.427
	FCM (10)	118.0 (90.7–168.0) [iU]/L		Within FCM: 0.016
**BALP**				
Baseline	FDI (14)	21.3 (16.6–26.8) [U]/L	0.462	
	FCM (12)	18.7 (14.1–27.5) [U]/L		
1–2 days post 1st infusion	FDI (14)	18.6 (15.7–26.7) [U]/L	0.767	
	FCM (11)	17.0 (12.6–31.8) [U]/L		
2 weeks	FDI (13)	20.4 (16.6–23.8) [U]/L	0.738	
	FCM (10)	17.9 (16.7–24.1) [U]/L		
1 month (2nd infusion)	FDI (12)	20.6 (15.0–24.2) [U]/L	0.740	
	FCM (11)	18.5 (17.1–35.4) [U]/L		
1–2 days post 2nd infusion	FDI (9)	20.9 (15.7–27.8) [U]/L	0.905	
	FCM (10)	19.9 (16.7–35.1) [U]/L		
2 months	FDI (12)	19.8 (17.6–25.0) [U]/L	0.203	Within FDI: 0.883
	FCM (10)	22.9 (19.9–28.8) [U]/L		Within FCM < 0.001
**CTx**				
Baseline	FDI (14)	0.84 (0.56–1.01) µg/ml	0.560	
	FCM (12)	0.98 (0.49–1.18) µg/ml		
1–2 days post 1st infusion	FDI (14)	0.81 (0.51–0.97 µg/ml	0.767	
	FCM (11)	0.73 (0.44–1.19) µg/ml		
2 weeks	FDI (13)	0.77 (0.55–0.87) µg/ml	0.927	
	FCM (10)	0.69 (0.51–1.05) µg/ml		
1 month (2nd infusion)	FDI (12)	0.74 (0.49–.105) µg/ml	0.316	
	FCM (11)	0.99 (0.58–1.32) µg/ml		
1–2 days post 2nd infusion	FDI (9)	0.71 (0.46–0.84) µg/ml	0.211	
	FCM (10)	0.88 (0.50–1.17) µg/ml		
2 months	FDI (12)	0.84 (0.50–1.13) µg/ml	0.582	Within FDI: 0.905
	FCM (10)	0.94 (0.60–1.48) µg/ml		Within FCM: 0.006
**P1NP**				
Baseline	FDI (14)	112.0 (70.3–178.8) µg/L	0.820	
	FCM (12)	103.0 (63.0–166.3) µg/L		
1–2 days post 1st infusion	FDI (14)	108.0 (63.6–156.8) µg/L	0.767	
	FCM (11)	89.0 (55.0–164.0) µg/L		
2 weeks	FDI (13)	107.0 (64.0–126.0) µg/L	0.784	
	FCM (10)	77.5 (56.3–158.8) µg/L		
1 month (2nd infusion)	FDI (12)	98.0 (63.0–118.0) µg/L	0.748	
	FCM (11)	85.0 (56.0–166.0) µg/L		
1–2 days post 2nd infusion	FDI (9)	80.0 (50.5–116.0) µg/L	0.780	
	FCM (10)	71.0 (56.7–174.5) µg/L		
2 months	FDI (12)	97.0 (50.8–156.5) µg/L	0.722	Within FDI: 0.439
	FCM (10)	104.5 (55.3–181.8) µg/L		Within FCM: 0.459

### Hematinic response, kidney function/injury and inflammation

Changes in hemoglobin, serum ferritin and transferrin saturation throughout the study for both groups were similar. Kidney function, proteinuria and markers of inflammation were not impacted by the use of intravenous iron (Supplementary table 4).

### Safety

Safety was monitored as part of pharmacovigilance throughout the study. A total of 8 serious adverse events occurred in 6 (23.1%) participants including one death (intestinal perforation). All of the serious adverse events were adjudicated as unrelated to the study drug. In total there were 19 adverse events, with possibly one on each group being related to the study group (mild hypophosphatemia) (Supplementary Table 5). Three (11.5%) participants in the FDI group commenced hemodialysis during the trial – two were unplanned and followed hospitalization and one was according to long-term planning (Supplementary Table 5).

## Discussion

The present study suggests that FCM and FDI have a differential effect on markers of 6H syndrome in patients with ND-CKD and iron deficiency with/without anemia. Significant changes in markers of bone/skeletal turnover, (ALP, BALP and CTx) were seen within the FCM group. Hematinic response and potential for kidney implications were similar between the two groups.

Ferric carboxymaltose was associated with a significant increase in iFGF23 within 1–2 days of administration, when compared to FDI. This lack of significant change for FDI has been demonstrated in previous randomized controlled trials in patients without kidney disease [9, 10]. The results were comparable with those of Huang and colleagues in their observational study of patients with ND-CKD receiving 1000 mg of FCM (*n* = 25, median eGFR: 32 ml/min/1.73m^2^) [23]. The repeat administration appeared to cause an enhanced increase in iFGF23, signaling a potentially primed system, a trend also shown in the PHOSPHARE-IDA randomized controlled trial [9]. The results correlate with previous studies examining the use of FCM, and suggest that in ND-CKD, even at low kidney function (median eGFR: 18 ml/min/1.73 m^2^), FCM appears to increase the concentration of bioactive iFGF23.

Phosphate decreased in patients following FCM administration when compared to those receiving FDI, as indicated by the significant difference at 2 weeks following the first iron administration. This period of 14 days coincides with the time-period over which nadir phosphate has been reported in other studies [8, 9]. In addition, based on the exploratory outcome of our study (a combined decrease of > 20% in phosphate and an increase of iFGF23 > 200%), which was only observed in the FCM group, the results suggest that the change in phosphate concentration may be partially explained by changes in FGF23 metabolism. It is important however to note that not all patients developing an increase in iFGF23 > 200% experienced the pre-determined decrease in phosphate of 20%, and this leads to the question of whether an iatrogenic increase in iFGF23 in this population may have other effects outside the scope of hypophosphatemia/phosphaturia. Indeed, there was no incidence of hypophosphatemia in the study as per the cut-off set (0.65 mmol/L), and no differential effect was noted in terms of fractional excretion of phosphate. One episode of transient, non-symptomatic mild hypophosphatemia was seen (< 0.81 mmol/L) in each group. The increasing trend in phophosphaturia in the FCM group and absence of hypophosphatemia may be explained partially by two mechanisms: iFGF23 resistance in the proximal convoluted tubule and the “intact nephron hypothesis”, whereby fractional excretion of phosphate is already high in patients with CKD [25, 26]. Klotho defiency, uremic toxins and direct tubular damage associated with albuminuria appear to contribute to resistance to FGF23. The low incidence of hypophosphatemia in the study, highlights certain difficulties with performing an adequately powered randomized controlled trial; however it allows for the formulation of further hypotheses whereby stratification of different grades of CKD and response to various intravenous iron formulations can be studied.

Fibroblast growth factor 23 leads to inhibition of 25(OH) vitamin D3 1a-hydroxylase and stimulation of 24-hydroxylase hence a reduction of the active form 1,25 (OH)_2_ Vitamin D [13]. In comparative randomized controlled trials both FCM and FDI have been shown to decrease 1,25 (OH)_2_ Vitamin D, with a more pronounced and longer-lasting decrease following FCM [9–11]. Metabolites of 1,25 (OH)_2_ Vitamin D also appear to increase following administration of FCM. In “Iron and Phosphatura – ExplorIRON-CKD”, a significant difference in effect was seen 1–2 days following 1st and 2nd infusions, indicating a decrease 1,25 (OH)_2_ Vitamin D in the FCM group. The concentrations of precursor and breakdown products 25(OH)_2_ Vitamin D and 24(R),25 (OH)_2_ Vitamin D were not significantly altered. This may be explained by the insufficient statistical power. Nonetheless observational studies using FCM in patients with ND-CKD have found similar results [23]. The novel finding of a nadir in 1,25 (OH)_2_ Vitamin D concentration 1–2 days following the 2nd administration of intravenous iron alongside an amplified % change, may suggest an accentuated effect with FCM. As CKD progression is associated with reduced vitamin D conferring an increased risk of mortality and mortality this finding warrants further research [27]. Indeed, the changes in 1,25 (OH)2 Vitamin D may be related to the trends in calcium concentration following administration of intravenous iron, with a negative trend noted in the FCM group. There was no change in PTH in contrast to other studies [9]. This could be due to the presence of secondary hyperparathyroidism in these participants with associated advanced CKD and this could confer a degree of resistance of the parathyroid glands to FGF23 [28]. The changes observed underline the potential of a differential effect dependent on the type of intravenous iron on FGF23 metabolism which also impacts on features of the 6H syndrome with the statistically significant difference in phosphate concentration, change in 1,25 (OH)_2_ Vitamin D and calcium, and the trend for increasing fractional excretion of phosphate exhibited here.

Only exposure to FCM led to a statistical significant changes in markers of bone mineralization (ALP and BALP) and bone resorption (CTx). This is intriguing, as numerically there was no detectable trends relevant to absolute concentration; however, in-vivo, both autosomal dominant hypophosphatemic rickets and x-linked hypophosphatemic rickets present with a phenotype similar to iatrogenic FCM induced hypophosphatemia with alterations in bone resorption and mineralization. These changes are associated with raised ALP and BALP, and the results of the comparative PHOSPHARE-IDA and PHOSPHARE-IBD are comparable to the present results [9, 11, 29, 30]. This indicates that the differential effect on FGF23 secondary to certain intravenous iron compounds may cause derangements in bone turnover and may have the potential to cause osteomalacia and fractures, especially after repeated infusions. Bone density has been previously shown to be associated with markers of bone turnover (especially BALP) in patients with kidney disease. It is important to highlight however that certain bone turnover markers such as CTx can be affected by kidney function [31].

Both intravenous iron compounds were associated with resolution of iron deficiency by the end of the trial period, with significant improvements in serum ferritin, transferrin saturation and hemoglobin concentration. Kidney function, proteinuria and inflammatory markers remained stable throughout the study, complementing the safety signals detected in FIND-CKD pertaining to FCM and other smaller studies relevant to FDI when used in patients with ND-CKD [32–34].

The “Iron and Phosphaturia – ExplorIRON-CKD” carries a number of inherent limitations. This was an exploratory study, with a small sample size. Not all patients contributed samples at all visits attended, and the intention-to-treat analysis allows for heterogeneity to be introduced. The randomization pool was composed by Caucasian individuals limiting generalizability to ethnic populations. There were no measurements of c-terminal FGF23 (assays binding on the c-terminal region of FGF23 detecting both cleaved and intact FGF23), therefore any interpretations of potential alterations in FGF23 metabolism secondary to FCM are limited only to the effect on iFGF23. Soluble klotho levels were also not measured during the study, hence limiting the identification of the pathophysiological cause of restricted phosphaturic effect throughout study. In addition, adjusted calcium was used as a surrogate marker of calcium concentration and not ionized calcium as per the KDIGO guidelines of CKD mineral bone disorder [35]. This may lead to underestimation of the effect of iron on calcium, as ionized calcium appears to be more “reactive” in PTH, and FGF23-associated pathologies [36, 37]. The data collection issues may have affected the statistical analysis especially within group, hence intergroup trends must be viewed with caution.

Nonetheless, the present exploratory study has identified signals which add important information for further hypothesis generation and trial design. The discussed results highlight the potential for a differential effect in terms of FGF23 and markers of the 6H syndrome in a setting consistent with clinical practice. In fact, the differential effect demonstrated in terms of FGF23 led to changes relevant to decrease in phosphate, calcium and vitamin D, in pattern and time intervals previously noted in other comparator trials. The findings relevant to bone turnover alongside amplified effects in terms of vitamin D, confer the potential of iatrogenic alterations secondary to FCM administration and warrant further research, especially when considering the frequency of intravenous iron utilization in this patient group. These features could be potentially elicited further in the future, through studies incorporating clinically relevant and objective markers of bone mineral density such as dual-energy X-ray absorptiometry or bone biopsy. On the other hand, the clinical response in terms of iron deficiency management accompanied by the lack of impact on kidney function further supplement the notion of safety and efficacy of modern intravenous iron compounds.

## Conclusions

The “Iron and Phosphaturia – ExplorIRON-CKD” trial was set up to explore the notion of the 6H syndrome in ND-CKD following administration of either FCM or FDI in patients with iron deficiency with/without anemia. A significant differential effect of iFGF23 was noted within 1–2 days following intravenous iron administration, due to the large increase recorded in the FCM group. This change was associated with a significant decrease in phosphate, 1,25 (OH)_2_ Vitamin D and calcium, but no effects on parathyroid hormone. Despite no apparent clinically relevant hypophosphatemia noted, the effects recorded on those markers alongside the impact on bone turnover require further research to ascertain the clinical implications of administration of certain intravenous iron compounds in patients with ND-CKD over a longer period and after repeated dosing.

### Supplementary Information

**Additional file 1.**

## Data Availability

The data associated with the paper are not publicly available due to sponsorship restrictions but are available from the corresponding author on reasonable request with the relevant permissions and agreement of the Research and Development Department of the Hull University Teaching Hospitals NHS Trust that served as the sponsor for the study. Further enquiries can be directed to the corresponding author.
