# Supplementary material for: The differential effect of modern intravenous iron on fibroblast growth factor 23 and phosphate in non-dialysis dependent CKD – the exploratory randomized controlled double-blind ExplorIRON-CKD study
Source: BMC Nephrol. 2024 Feb 12;25:54. doi: 10.1186/s12882-023-03440-7 (PMC10860218; doi:10.1186/s12882-023-03440-7)
Supplement: Supplementary file 1 — Additional file 1. [file 12882_2023_3440_MOESM1_ESM.docx]

**Supplementary Materials:**

| **Supplementary table 1: Inclusion / Exclusion criteria** | |
| --- | --- |
| **Inclusion** | **Exclusion** |
| - Men and women aged ≥18 years | - Pregnancy or lactation |
| - Patients with CKD stages 3a-5 (not on dialysis) | - Patients being investigated for potential blood loss |
| - Resting blood pressure ≤ 160/95mmHg | - Dialysis patients (either peritoneal or hemodialysis) |
| - Able to give written and signed informed patient consent | - Weight ≤ 70kg; if hemoglobin is ≥ 100 g/L; |
| - Able to complete study assessments | - Bleeding (> 500 ml) or surgery in the 30 days prior to recruitment |
| - Ferritin level less than 200µg/L OR transferrin saturation ≤20% and serum ferritin between 200-299µg/L | - Known allergy to iron therapy |
|  | - Symptomatic ischemic heart disease |
| - Hemoglobin < 150g/L | - Hemochromatosis or history of acquired iron overload |
| - Serum phosphate > 0.8 mmol/L | - Parenteral iron therapy or red blood cell transfusion within the previous 6 weeks; |
| - A negative pregnancy test for females of child bearing potential | - Inability to co-operate with study protocol |
| - Suitable contraception for females of child bearing potential for duration of study | - Active infection or a CRP>50 mg/L where clinical suspicion arises |
|  | - Patients with potential confounding factors - cancer, (with exception of basal cell or squamous cell carcinoma of the skin, and cervical intraepithelial neoplasia) |
|  | - Patients who are unable or do not wish to give consent |
|  | - Patients with known hemoglobinopathy, myelodysplasia, myeloma |
|  | - Involvement in another clinical trial of an investigational medicinal product within the past four weeks |
| As it appears in: Kassianides X, Bhandari S. Methodology and Baseline Data of a Comparative Exploratory Double-Blinded Randomized Study of Intravenous Iron on Fibroblast Growth Factor 23 and Phosphate in Chronic Kidney Disease. Kidney Blood Press Res. 2023 Apr 4;48(1):151-164. doi: 10.1159/000528313. Epub ahead of print. PMID: 37015198. | |

| **Supplementary table 2: Study outcomes** | |
| --- | --- |
| Outcome | Domain |
| Primary outcome | |
| Percentage (%) change in iFGF23 from baseline to 1-2 days post-infusion between FDI and FCM | FGF-23, phosphate and bone metabolism |
| Co-primary outcome (exploratory – *no statistical analysis*) | |
| Composite of change in iFGF23 and delta change in phosphate at 2 days and 2 weeks. | FGF-23, phosphate and bone metabolism |
| Pre-specified Secondary outcomes | |
| % change in iFGF23 from baseline to 2 weeks post-infusion between FDI and FCM | FGF-23, phosphate and bone metabolism |
| Difference between the two treatments in terms of 6H markers | FGF-23, phosphate and bone metabolism |
| Difference between the two treatments in terms of bone turnover markers | FGF-23, phosphate and bone metabolism |
| Incidence of hypophosphatemia (<0.65 mmol/L) and severe hypophosphatemia (<0.3mmol/L) at each time point | FGF-23, phosphate and bone metabolism |
| % failed repeat infusion due to hypophosphatemia | FGF-23, phosphate and bone metabolism |
| Difference in the co analysis of clinical end points including hematinic and kidney function variables | Clinical measures |
| Adapted from Kassianides X, Bhandari S. Methodology and Baseline Data of a Comparative Exploratory Double-Blinded Randomized Study of Intravenous Iron on Fibroblast Growth Factor 23 and Phosphate in Chronic Kidney Disease. Kidney Blood Press Res. 2023 Apr 4;48(1):151-164. doi: 10.1159/000528313. Epub ahead of print. PMID: 37015198. | |

| **Supplementary table 3: Reference ranges of variables** | |
| --- | --- |
| **Variable** | **Reference range** |
| iFGF23 / pg/ml | 28.0-121.0 |
| Phosphate / mmol/L | 0.80–1.50 |
| Hemoglobin / g/L | > 120.0 |
| Serum Ferritin / μg/L | > 100.0 |
| Transferrin saturation / % | > 20% |
| Creatinine / μmol/L | N/A |
| eGFR / ml/min/1.73m2 | N/A |
| CRP / mg/L | < 8.0 |
| urinary PCR / mg/mmol | < 50.0 |
| 24hr urinary phosphate / mmol/24hr | 16.0-48.0 |
| FEP / % | < 20% |
| Calcium / mmol/L | 2.20 – 2.60 |
| PTH / pmol/L | 1.30-9.30 |
| 1,25 (OH)_2_ Vitamin D / pmol/L | 48.0-150.0 |
| 25 (OH)_2_ Vitamin D / nmol/L | N/A |
| 24(R),25 (OH)_2_ Vitamin D / nmol/L | N/A |
| ALP / [iU]/L | 30.0-125.0 |
| BALP / [U]/L | Male:15.0-41.3  Post-menopause Female: 14.2–42.7 |
| CTx / μg/ml | 0.10-0.50 |
| P1NP / μg/L | 26.0-110.0 |

| **Supplementary table 4: Hematinic response and markers of kidney function/injury and inflammation** | | | | | | | | | |
| --- | --- | --- | --- | --- | --- | --- | --- | --- | --- |
| **Visit** | **Iron group (n)** | **Mean/Median (SD/IQR)** | **p-value** | **p-value (within group)** | **Visit** | **Iron group (n)** | **Mean/Median (SD/IQR)** | **p-value** | **p-value (within group)** |
| **Hemoglobin * / g/L** | | | | | **eGFR / ml/min/1.73m^2^** | | | | |
| Baseline | FDI (14) | 99.2 (12.2) | 0.664 |  | Baseline | FDI (14) | 18.0 (14.0-25.3) | 1.000 |  |
|  | FCM (12) | 101.6 (15.3) |  |  |  | FCM (12) | 18.0 (14.0-25.3) |  |  |
| 2 weeks | FDI (13) | 105.2 (9.5) | 0.645 |  | 2 weeks | FDI (13) | 21.0 (13.0-25.5) | 0.738 |  |
|  | FCM (10) | 102.7 (16.3) |  |  |  | FCM (10) | 18.5 (15.0-26.8) |  |  |
| 1 month | FDI (12) | 103.8 (13.0) | 0.707 |  | 1 month | FDI (12) | 19.5 (12.5-23.0) | 0.880 |  |
|  | FCM (11) | 106.0 (14.3) |  |  |  | FCM (11) | 19.0 (14.0-28.0) |  |  |
| 2 months | FDI (13) | 106.0 (13.3) | 0.526 | Within FDI: 0.041 | 2 months | FDI (13) | 19.0 (11.5-25.5) | 1.000 | Within FDI: 0.811 |
|  | FCM (10) | 109.1 (8.3) |  | Within FCM: 0.002 |  | FCM (10) | 16.5 (13.8-22.3) |  | Within FCM: 0.726 |
| **Creatinine * / μmol/L** | | | | | **urinary PCR / mg/mmol** | | | | |
| Baseline | FDI (14) | 277.6 (98.8) | 0.626 |  | Baseline | FDI (13) | 155.0 (57.5-607.5) | 0.082 |  |
|  | FCM (12) | 260.2 (77.3) |  |  |  | FCM (11) | 30.0 (20.0-310.0) |  |  |
| 2 weeks | FDI(13) | 278.2 (106.0) | 0.570 |  | 2 weeks | FDI (11) | 85.0 (60.0-325.0) | 0.056 |  |
|  | FCM (10) | 256.4 (61.0) |  |  |  | FCM (9) | 20.0 (15.0-107.5) |  |  |
| 1 month | FDI (12) | 275.3 (95.1) | 0.526 |  | 1 month | FDI (10) | 130.0 (60.0-517.5) | 0.085 |  |
|  | FCM (11) | 252.4 (72.4) |  |  |  | FCM (11) | 30.0 (15.0-145.0) |  |  |
| 2 months | FDI (13) | 288.6 (100.9) | 0.638 | Within FDI: 0.527 | 2 months | FDI (10) | 157.5 (53.7-585.0) | 0.089 | Within FDI: 0.663 |
|  | FCM (10) | 271.0 (65.9) |  | Within FCM: 0.421 |  | FCM (10) | 35.0 (17.5-191.3) |  | Within FCM: 0.793 |
| **Serum ferritin / μg/L** | | | | | **Transferrin saturation / %** | | | | |
| Baseline | FDI (14) | 76.5 (25.0-183.5) |  |  | Baseline | FDI (14) | 15.0 (11.0-21.0) | 0.781 |  |
|  | FCM (12) | 72.7 (42.3-146.9) | 0.899 |  |  | FCM (12) | 14.5 (12.0-17.8) |  |  |
| 1-2 days post 1st infusion | FDI (14) | 190.5 (132.7-223.0) |  |  | 1-2 days post 1st infusion | FDI (14) | 100.0 (91.0-100.0) | 1.000 |  |
|  | FCM (11) | 231.0 (169.0-377.0) | 0.107 |  |  | FCM (11) | 100.0 (88.0-100.0) |  |  |
| 2 weeks | FDI (13) | 344.0 (249.5-472.0) |  |  | 2 weeks | FDI (13) | 22.0 (17.5-34.0) | 0.605 |  |
|  | FCM (10) | 483.0 (325.8-723.8) | 0.115 |  |  | FCM (10) | 25.5 (18.8-32.8) |  |  |
| 1 month (2^nd^ infusion) | FDI (12) | 299.5 (254.0-442.8) |  |  | 1 month (2^nd^ infusion) | FDI (12) | 26.0 (17.0-30.8) | 0.740 |  |
|  | FCM (11) | 318.0 (276.0-515.0) | 0.740 |  |  | FCM (11) | 22.0 (17.0-30.0) |  |  |
| 1-2 days post 2^nd^ infusion | FDI (9) | 331.0 (286.5-383.0) |  |  | 1-2 days post 2^nd^ infusion | FDI (9) | 89.0 (62.0-100.0) | 0.161 |  |
|  | FCM (10) | 409.5 (327.5-609.8) | 0.156 |  |  | FCM (9) | 67.0 (58.0-78.0) |  |  |
| 2 months | FDI (13) | 406.0 (349.5-472.0) |  | Within FDI <0.001 | 2 months | FDI (13) | 29.0 (22.5-32.0) | 0.927 | Within FDI <0.001 |
|  | FCM (10) | 415.5 (351.8-605.3) | 0.563 | Within FCM <0.001 |  | FCM (10) | 26.0 (20.5-38.0) |  | Within FCM <0.001 |
| **CRP / mg/L** | | | | |  | | | | |
| Baseline | FDI (14) | 8.0 (3.2-20.8) | 0.462 |  |  |  |  |  |  |
|  | FCM (12) | 4.3 (3.4-13.4) |  |  |  |  |  |  |  |
| 2 weeks | FDI (13) | 7.7 (2.9-23.5) | 0.738 |  |  |  |  |  |  |
|  | FCM (10) | 6.4 (5.2-23.3) |  |  |  |  |  |  |  |
| 1 month | FDI (12) | 8.0 (1.9-17.0) | 0.651 |  |  |  |  |  |  |
|  | FCM (11) | 4.9 (4.5-23.0) |  |  |  |  |  |  |  |
| 2 months | FDI (13) | 8.6 (1.6-29.5) | 0.832 | Within FDI: 0.456 |  |  |  |  |  |
|  | FCM (10) | 6.2 (4.7-16.7) |  | Within FCM: 0.114 |  |  |  |  |  |
| * variables characterized by asterisk are described as mean (SD); the remaining variables are described as median (IQR) based on distribution | | | | | | | | | |

| **Supplementary table 5: Safety profile** | | |
| --- | --- | --- |
| **Adverse event** | **Medication** | **SAE** |
| Infected liver cyst (leading to dialysis) | FDI | x |
| Atrial fibrillation | FDI | x |
| Vacant episode | FDI | x |
| Dialysis Initiation | FDI | x |
| Hyponatremia/hypokalemia | FDI | x |
| Infected rectal stump | FDI | x |
| Acute cholecystitis | FCM | x |
| Acute intestinal perforation | FCM | x |
| Nausea | FDI |  |
| Diarrhea | FDI |  |
| Initiation of dialysis | FDI |  |
| Urinary tract infection | FDI |  |
| Lower limb cellulitis | FDI |  |
| Gout | FDI |  |
| Mild hypophosphatemia | FDI |  |
| Urinary tract infection | FDI |  |
| Asymptomatic rise in CRP | FDI |  |
| Presyncope | FDI |  |
| Neck stiffness/pain | FDI |  |
| Pleurisy | FCM |  |
| Urinary tract infection | FCM |  |
| Otitis media | FCM |  |
| Mild hypophosphatemia | FCM |  |
| Urinary tract infection | FCM |  |
| Urinary tract infection | FCM |  |
| Cellulitis | FCM |  |
| Gout | FCM |  |
| Total SAEs: 8 in 6 patients (FDI: 4; FCM: 2)  Total AEs: 27 in 18 patients (FDI: 12; FCM: 8)  All SAEs required hospitalization | | |
